# Endoscopic lithotripsy for Bouveret syndrome complicated by small bowel obstruction secondary to gallstone fragments

**DOI:** 10.1093/jscr/rjab118

**Published:** 2021-04-19

**Authors:** Dulani Goonawardhana, Roy Huynh, Joel Rabindran, Guillermo Becerril-Martinez

**Affiliations:** Department of Upper Gastrointestinal Surgery, Concord Repatriation General Hospital, Hospital Rd, Concord, New South Wales, Australia; Department of Upper Gastrointestinal Surgery, Concord Repatriation General Hospital, Hospital Rd, Concord, New South Wales, Australia; Department of Upper Gastrointestinal Surgery, Concord Repatriation General Hospital, Hospital Rd, Concord, New South Wales, Australia; Department of Upper Gastrointestinal Surgery, Concord Repatriation General Hospital, Hospital Rd, Concord, New South Wales, Australia

## Abstract

Bouveret syndrome is a rare complication of cholecystitis, in which impaction of a gallstone creates a cholecystoduodenal fistula leading to gastric outlet obstruction. We report a case of a 90-year-old female who presented with nausea and vomiting on a background of previous necrotic cholecystitis managed conservatively. Computed tomography of the abdomen demonstrated a large gallstone impacted in the third part of the duodenum leading to gastric outlet obstruction. Given her frailty, the patient underwent endoscopy to relieve the obstruction; however, complete retrieval of the gallstone fragments after lithotripsy was not possible. She subsequently developed distal gallstone ileus due to migration of the gallstone fragments and underwent laparotomy, enterotomy and retrieval of the fragments. This case highlights the dilemma of managing elderly patients with Bouveret syndrome with open or endoscopic surgery and the importance of retrieving all gallstone fragments after lithotripsy to avoid iatrogenic complications, such as gallstone ileus.

## INTRODUCTION

Bouveret syndrome is a rare complication of cholecystitis, where impaction of a large gallstone through a cholecystoduodenal fistula into the proximal duodenum leads to gastric outlet obstruction (GOO) [1]. Gallstone ileus only makes up 1–4% of bowel obstructions and Bouveret syndrome comprises just 3% of these cases [2]. It most commonly affects the older population with multiple medical comorbidities, with an average age of 74 years at the time of onset [3]. Consequently, it is associated with high morbidity and mortality risks, approximately 60% and 12–30%, respectively [4]. We discuss a case of Bouveret syndrome initially managed with endoscopic lithotripsy, however, subsequently requiring open surgical management after the development of distal gallstone ileus.

## CASE REPORT

**Figure 1:**
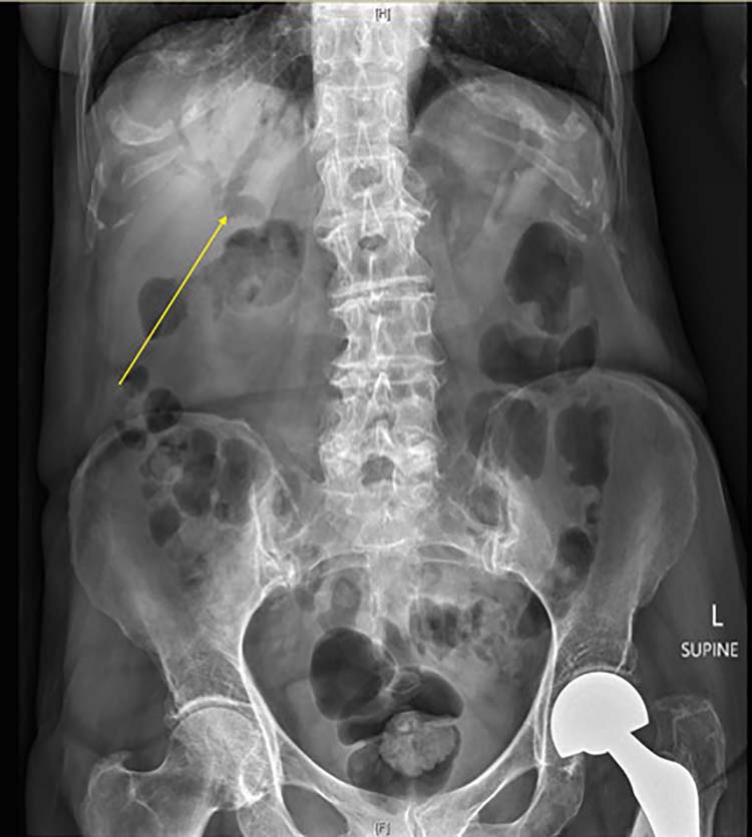
Abdominal X-ray with arrow demonstrating pneumobilia.

**Figure 2:**
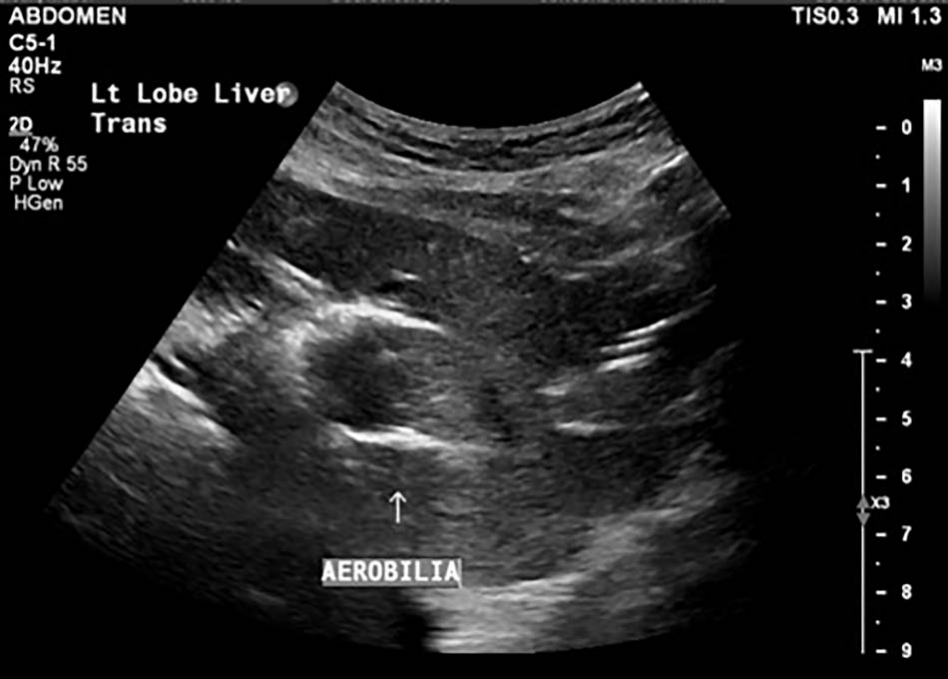
Abdominal ultrasound demonstrating pneumobilia.

**Figure 3:**
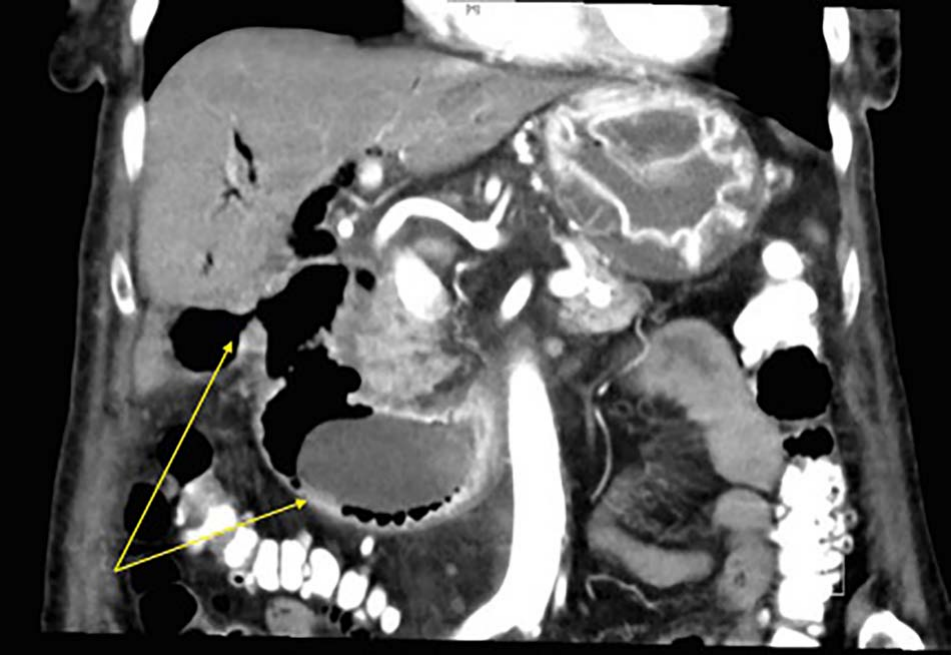
Coronal CT image of abdomen with arrows demonstrating a fistulous connection between the gallbladder and duodenum, and a large gallstone in the second part of the duodenum.

A 90-year-old female presented to the Emergency Department with right upper quadrant pain associated with nausea and persistent vomiting. She had been admitted seven months prior with acute necrotic cholecystitis, but given her advanced age, frailty, medical comorbidities and wishes to avoid surgery, she was managed with intravenous antibiotics and percutaneous cholecystostomy, which was removed eight weeks later. The patient was haemodynamically stable on presentation, but on examination had percussion tenderness in the right upper quadrant. Both abdominal X-ray and ultrasound demonstrated pneumobilia (Figs 1 and 2). A computed tomography (CT) of the abdomen showed a cholecystoduodenal fistula with a 60 × 30-mm gallstone in the third part of the duodenum causing gastric outlet obstruction (GOO), consistent with Bouveret syndrome (Fig. 3).

**Figure 4:**
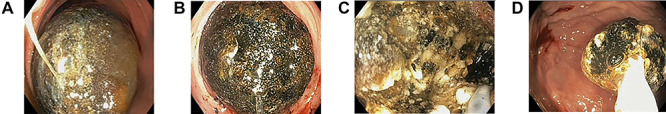
(**A**) Large gallstone in the second part of the duodenum on endoscopy causing complete obstruction, (**B**) minimal effect of electrohydraulic lithotripsy seen on the gallstone, (**C**) breakdown of the gallstone using Holmium laser lithotripsy, (**D**) mobilization of gallstone fragment into pylorus after lithotripsy.

The patient was offered a laparotomy and retrieval of the gallstone via gastrotomy, however, she declined. Given her wishes for non-operative management, endoscopic treatment was offered to relieve her persistent GOO. Endoscopy corroborated a cholecystoduodenal fistula in the duodenal cap and complete obstruction with the gallstone impacted in the second to third part of the duodenum. Retrieval of the gallstone was attempted with a 3-cm trapezoid wire-guided retrieval basket, but the size of the stone prevented capture. Electrohydraulic lithotripsy was then performed with minimal effect due to the hard consistency of the gallstone. Holmium laser lithotripsy was utilized, which achieved fragmentation of the stone. The duodenal obstruction was resolved and the larger fragments were mobilized into the stomach (Fig. 4A–D). Given the lengthy operative time, and no availability of a basket to retrieve the fragments, a planned second-stage endoscopic procedure was decided.

**Figure 5:**
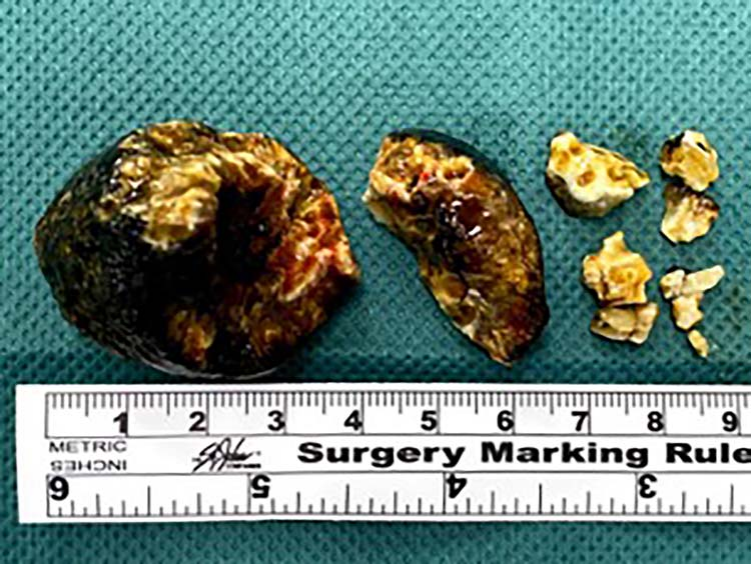
Multiple gallstone fragments retrieved during laparotomy measuring up to 4 cm in diameter.

**Figure 6:**
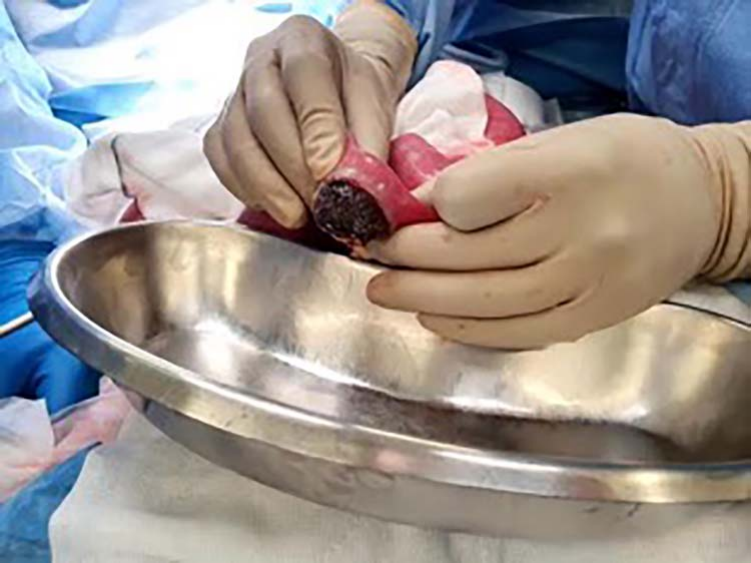
Large gallstone fragment retrieved through enterotomy in the small bowel.

Post-operatively, the patient was monitored in the intensive care unit. Despite the intervention, her nasogastric output increased and she developed obstipation. A progress CT abdomen and pelvis revealed a small bowel obstruction secondary to a gallstone fragment in the left lower quadrant. Ongoing conservative management with bowel rest, gastric decompression and total parenteral nutrition did not improve the patient’s state. Following discussions with the patient and family, she underwent laparotomy, which demonstrated a transition point 7 cm from the ileocaecal valve where an adhesion from previous hysterectomy was noted. A 4-cm gallstone fragment along with multiple other fragments was found and retrieved through a longitudinal enterotomy on the anti-mesenteric border (Figs 5 and 6). The enterotomy was then closed primarily and patched with omentum. Intraoperative gastroscopy did not find any residual fragments in the stomach.

Post-operatively, the patient progressed slowly due to frailty, ileus and malnutrition. She was transferred to the geriatrics rehabilitation unit following resolution of her surgical issues. She has since been discharged into the community and was well at our last review.

## DISCUSSION

Bouveret syndrome is a rare, but highly morbid condition largely affecting the elderly population [3, 4]. Given the rare occurrence of Bouveret syndrome, there are currently no standardized recommendations for patient management. The most common management is surgery with gastrotomy with or without duodenotomy and stone retrieval. Despite this, the frailty of patients with this condition must be accounted for, and so, endoscopic management is often utilized in the first instance [4].

Endoscopic retrieval using baskets and nets can be utilized, however, are ineffective in removing large stones, as seen in this case. Adjuncts such as lithotripsy are often required to break the stone into fragments that are amenable to retrieval. Techniques include mechanical, electrohydraulic, laser or extracorporeal shockwave lithotripsy [5]. Laser lithotripsy is a promising technique as it targets energy into the stone and reduces surrounding tissue injury. The limitations of endoscopic therapy include requiring a skilled endoscopist, difficulty visualizing the stone and often, the need for multiple modalities of treatment [5]. Furthermore, care must be taken to ensure fragments are not dislodged distally and patients should be monitored post-procedure to ensure a distal gallstone ileus does not develop [6, 7].

We report a case of Bouveret syndrome managed with endoscopic lithotripsy to relieve duodenal obstruction with secondary gallstone ileus due to fragments. This case highlights the clinical dilemma surgeons face between endoscopic and open surgical management of Bouveret syndrome, which was complicated by the co-morbidities and prior surgical history in this patient. Furthermore, we emphasize the importance of immediate retrieval of all gallstone fragments following lithotripsy to prevent distal gallstone ileus, particularly in those with previous lower abdominal surgery.

## CONFLICT OF INTEREST STATEMENT

There is no conflict of interest to disclose.

## FUNDING

No funding was sought.
